# How Does the Use of an Intraoral Scanner Affect Muscle Fatigue? A Preliminary In Vivo Study

**DOI:** 10.3390/bioengineering9080358

**Published:** 2022-08-01

**Authors:** KeunBaDa Son, Ji-Min Lee, Young-Tak Son, Jin-Wook Kim, Myoung-Uk Jin, Kyu-Bok Lee

**Affiliations:** 1 Advanced Dental Device Development Institute (A3DI), Kyungpook National University, Daegu 41940, Korea; oceanson@knu.ac.kr (K.S.); wlals9408@naver.com (J.-M.L.); dudxkr741@naver.com (Y.-T.S.); 2 Department of Dental Science, Graduate School, Kyungpook National University, Daegu 41940, Korea; 3 Department of Conservative Dentistry, School of Dentistry, Kyungpook National University, Daegu 41940, Korea; 4 Department of Oral & Maxillofacial Surgery, School of Dentistry, Kyungpook National University, Daegu 41940, Korea; 5 Department of Prosthodontics, School of Dentistry, Kyungpook National University, Daegu 41940, Korea

**Keywords:** dentistry, dental unit chair systems, muscle fatigue, muscle activation, in vivo study

## Abstract

The purpose of this study was to evaluate muscle activation and fatigue in the operator during tooth preparation and intraoral scanning by simulating these tasks in two types of dental unit chair systems (UCS). Six participants were recruited, and the above tasks were simulated. Electrodes were placed on the skin over five types of muscles (arm, neck, and shoulder muscles), and the maximal voluntary contraction (*MVC*) was measured. Electromyography (EMG) was assessed during the simulation, and EMG values were normalized using *MVC*. The root mean square (RMS) EMG (%*MVC*) and muscle fatigue (%) were calculated. Owing to a lack of normal distribution of the data, Mann–Whitney U test and Kruskal–Wallis H test were performed for statistical comparison, and Bonferroni adjustment was performed for multiple comparisons (α = 0.05). There was no significant difference in *RMS EMG* between the two types of dental UCS (intraoral scanning, *p* = 0.237; tooth preparation, *p* = 0.543). Moreover, the *RMS EMG* and muscle fatigue were not significantly different between the two tasks (*p* > 0.05). There was significant muscle fatigue after the intraoral scanner use was simulated thrice (*p* < 0.001). It is necessary to refrain from performing continuous intraoral scanning and tooth preparation and to take appropriate rest to reduce the incidence of musculoskeletal disorders in dentists in clinical settings.

## 1. Introduction

In dental clinical practice, the use of a dental unit chair system (UCS) is essential for patient diagnosis and treatment [1,2]. Dentists spend most of their work time in the dental UCS for patient care [3]. The dental UCS consists of an operating light and a patient seat, foot controller, water fountain and cuspidor, monitor, bracket table, and dentist’s chair [4]. In addition, the dental UCS has been developed to facilitate the use of various dental medical devices and treatment tools [5,6].

Musculoskeletal disorders (MSDs) frequently occur among dental practitioners [7]. It is very difficult for a dentist to adopt an optimal working position because of the limited working space and long duration of treatment [8]. In addition, a high degree of concentration is required by the dentist during treatment resulting in a static posture being maintained for a long time [9]. In the process of maintaining a static posture, the parts of the dentist’s body most affected are the back, shoulders, and neck [10,11].

Electromyography (EMG) is a method for measuring electrical signals generated in the skeletal muscles to quantitatively evaluate the magnitude of muscle fatigue or exerted strength [9,10,11,12]. Since EMG evaluation can diagnose the functional abnormalities of muscles, it is widely used in various fields, such as medical research, rehabilitation medicine, sports science, and design engineering [9,10,11,12]. Muscle fatigue refers to a temporary decrease in the ability of a muscle or muscle group to generate force or perform physical activity and is an essential factor affecting working efficiency [10,11,12]. Therefore, muscle fatigue is highly correlated with muscle EMG activity and the root mean square (RMS) of EMG [10,11]. 

Several muscle groups, including the arms, neck, shoulders, and back, are activated during dental work. The arm muscles, flexor digitorum superficialis (FDS), and extensor digitorum communis (EDC) are activated during bending of the wrist and application of force for gripping dental instruments [13]. The sternocleidomastoid muscle (SCM) is involved when turning the head, and the splenius capitis (SC) is involved when bending the head to observe the patient’s mouth [9,10,12,13,14,15]. The trapezius descendens (T), which is used to raise the shoulder, has also been frequently used for assessment of EMG in dentists [9,10,12,13,14,15]. Therefore, it is important to reduce or prevent MSDs in the aforementioned muscles. There are several examples of application of ergonomics in dentistry, including in the patient chair, operator chair, operating light, hand instrumentation, and cabinetry.

Recently, as the application of dental computer-aided design and computer-aided manufacturing (CAD/CAM) has rapidly increased. The use of intraoral scanners has also increased [16]. Although manufacturers have reduced the weight and size of intraoral scanners for usability, these scanners are still one of the heaviest medical devices used directly in the oral cavity [17]. The weight of the intraoral scanner suggested by the manufacturer generally ranges from 113 g to 585 g; the scan time is more than five minutes per complete arch and the device is used repeatedly [17,18]. Although studies have reported the evaluation of EMG when a dentist performs tooth preparation using a high-speed handpiece [9,11], there have been no reports on the effect of intraoral scanner use on the dentist’s MSDs.

There is a need for further research on muscle activity and fatigue considering MSDs in various dental practices. Therefore, the purpose of this study was to evaluate muscle activation and fatigue in the operator during tooth preparation and intraoral scanning by simulating these two tasks in the two types of dental UCS. The null hypothesis of this study was that there is no significant difference in muscle activity and fatigue between the two types of dental UCS and the two types of tasks (tooth preparation and intraoral scanning). Additionally, we hypothesized that there is no difference in muscle activity and fatigue caused by repeated use of the intraoral scanner.

## 2. Materials and Methods

### 2.1. Participants

This clinical trial was approved by the Clinical Trial Ethics Committee of Kyungpook National University Dental Hospital (IRB No. KNUDH-2021-04-04-00). Right-handed participants with no history of MSDs were recruited. The study inclusion criteria specified that individuals with right-handedness or who presented with musculoskeletal disorders were excluded. The study exclusion criteria specified that individuals with musculoskeletal disorders, sensory or mental abnormalities, debilitating medical conditions, and/or who were pregnant, or lactating were not eligible for assessment in this study. For blinding, all participants did not know the purpose of the present study, and the experiment was performed only according to the instructions of one investigator. The sample size was calculated as at least four participants per group based on the results of a previous study [10] (G*Power version 3.1.9.2; Heinrich-Heine-Universität Düsseldorf, Düsseldorf, Germany) (actual power = 99.11%; power = 95%; α = 0.05); the present study included six participants per group. The mean age of the participants was 31.5 ± 3.9 years. The participants had a mean height of 170 ± 6.2 cm, mean weight of 66.3 ± 10 kg, and a dental clinical experience of 3.6 ± 1.1 years. The six participants consisted of two women and four men.

### 2.2. Data Collection: Ag/AgCl Electrode Placement on Sampled Muscles

**Figure 1:**
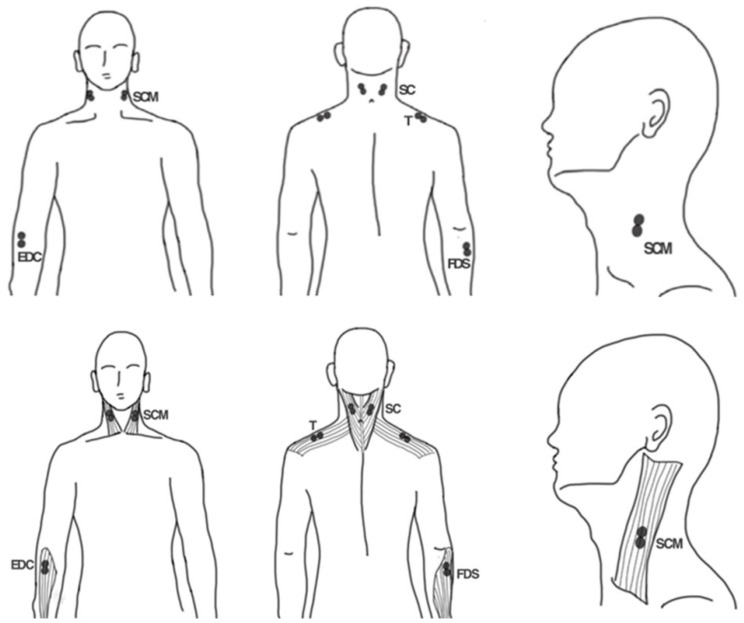
Schematic of the electrode attachment position for electromyography. EDC, extensor digitorum communis; FDS, flexor digitorum superficialis; SCM, sternocleidomastoid muscle; SC, splenius capitis; T, trapezius descendens.

The present study refers to the location for evaluation of MSDs that develop during dental treatment in the dental UCS as observed in previous studies [6,7,8,9,10,11]. The muscles to be assessed for surface EMG, EDC, and FDS were the arm muscles; neck muscles (SCM and SC); and shoulder muscle (T) (Figure 1). For the arm muscles, a pair of 20 mm diameter silver or silver chloride solid adhesive pre-gelled electrodes (Covidien, Mansfield, MA, USA) were attached only to the right hand to perform the task (Figure 1). For the other muscles, the electrodes were symmetrically attached to the left and right sides (Figure 1). Before attaching the electrode, the attachment site was made free of excess hair and thoroughly washed with a 70% isopropyl alcohol swab. According to the guidelines of the surface electromyography for the non-invasive assessment of muscles (SENIAM) protocol for each muscle location, two electrodes were attached to the movement point of each muscle in the direction of the muscle fiber [19]. The center distance between the two electrodes was 20 mm, and the ground electrode was attached to the sphenoid process of the left ulna (Figure 1) [19].

For the EDC, the electrodes were attached to the quarter point between the lateral epicondyle of the humerus and the styloid process of the ulna (Figure 1) [20,21]. For the FDS, the electrodes were attached to the quarter of the medial border of the medial epicondyle of the humerus and the coronoid process of the ulna (Figure 1) [20]. For the SCM, the electrodes were attached at the third point between the mastoid process and the sternal notch toward the sternal portion of the muscle [20]. For the SC, the electrodes were attached to the midpoint between the mastoid process and vertebra C7. For the T, the electrodes were attached to the midpoint between the acromion and vertebra C7 (Figure 1) [20].

After electrode placement, the electrode was connected to an EMG measuring system (WEMG-8; LAXTHA, Daejeon, Korea). In the measurement system, each channel was amplified to 244 µV through the EMG preamplifier, and the analog and digital signals were converted to a 10-bit resolution through the AD converter. The sample was collected at a sampling rate of 1024 Hz. Real-time EMG measurement software (TeleScan ver 3.29; LAXTHA, Daejeon, Korea) was used to collect real-time EMG data. 

### 2.3. Data Collection: Maximal Voluntary Contraction (MVC) Measurement

To normalize the EMG data, *MVC* was measured according to the guidelines of the SENIAM protocol [11,21]. All *MVC* measurements were performed while sitting on a dentist’s chair and supporting the lower back on the backrest. When measuring the *MVC* of the arm muscles, the forearm was supported on a desk and the elbow was bent at 90°. The EDC was measured by providing the maximum resistance force when opening the back of the hand and fingers, and the FDS measured the force to maximally close the fingers and palms using a grip force meter. The SCM was measured while providing the maximum resistance to the left and right rotations of the head with both arms lowered. The shoulder muscle (T) was measured by providing the maximum resistance force when trying to lift the shoulder upward. Each muscle was assessed three times at 5 s intervals, and the highest value was defined as the *MVC*.

### 2.4. Data Collection: Muscle Activation Measurement

**Figure 2:**
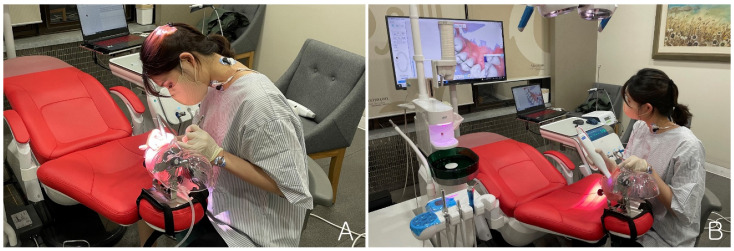
Electromyography measurements during dental simulations. (**A**) Tooth preparation simulation; (**B**) Intraoral scanning simulation.

After taking a break for 30 min after the *MVC* measurement, dental work simulations were performed on a dental mannequin (Simple Manikin III, NISSIN, Kyoto, Japan) installed in the dental UCS, and muscle activity was recorded in eight EMG channels. The participants performed simulations for intraoral scanning and tooth preparation tasks for two days at intervals of one week to prevent fatigue accumulation between tasks, and the work order was randomly selected by listing all orders (Figure 2).

The digital integrated dental UCS (MEGAGEN, Daegu, Korea) used an intraoral scanner (i500; MEDIT, Seoul, Korea) and monitored the dental UCS, and the conventional dental UCS (Maxpert; SHINHUNG, Seoul, Korea) showed the scanning process on a separate monitor, other than that of the dental UCS, connected to an intraoral scanner. The participants performed all work procedures after adjusting the dentist’s chair and the patient’s chair to fit their posture and body.

The intraoral scanning task was performed by consecutively scanning the maxillary and mandibular models for dental education (D85DP-500B.1; Nissin Dental, Kyoto, Japan) three times using an intraoral scanner (i500; MEDIT, Seoul, Korea; Figure 2). The scanning strategy was to scan the complete arch in the order of occlusal, buccal, and lingual, and all participants performed a scan so that there were no empty spaces in any of the teeth (Figure 2). The weight of an intraoral scanner used in the present study was 280 g.

The tooth preparation task was performed by preparing the maxillary right first molar (D85DP-500B.1; Nissin Dental, Kyoto, Japan) for a single ceramic crown and chamfer margin using a high-speed dental handpiece (TG-98; W&H, Bürmoos, Austria; Figure 2). Participants performed the tooth preparation task without a magnification system (Figure 2).

One investigator (J.M.L.) recorded the muscle activity in real time only when the participant performed any action for the tasks and did not record the muscle activity unless the participant performed the simulation. In addition, all working times were recorded.

### 2.5. Data Collection: Muscle Activation Analysis

Muscle activation and muscle fatigue were calculated from the data measured using *EMG* measurement software (TeleScan ver 3.29; LAXTHA, Daejeon, Korea). *EMG* data from dental work were normalized and expressed as percentages, and the activation of each muscle was calculated as follows [9,12] (1):

(1) $$RMS\;EMG\left(\%MVC\right)=\frac{Muscle\;activation\;during\;tasks\;\left(µV\right)}{MVC}\times 100$$

*RMS EMG* (%*MVC*) indicates muscle activation that occurs during dental work compared to MVC. As the *RMS EMG* (%*MVC*) increased, the risk of MSDs increased, and the ergonomic risk level according to the activation level of each muscle was evaluated according to previous literature: *MVC* in the range of 0–10% means “low risk”; 11–20% means “moderate risk,” and more than 21% means “high risk” [9,12,13].

Muscle fatigue can be identified by increasing and decreasing median edge frequency (*MEF*) values, and as *MEF* decreases, muscle fatigue increases [22,23,24,25]. The *MEF* value can be obtained in the frequency range of 1–400 Hz after applying the fast Fourier transform, which transforms the *EMG* signal that changes with time into a frequency. Among the total working time, *MEF* in the first 60 s and next 60 s were calculated, and muscle fatigue was calculated according to the following formula [23,24] (2):

(2) $$Muscle\;fatigue\left(\%\right)=\frac{MEF\;in\;the\;second\;60\;s-MEF\;in\;the\;first\;60\;s}{MEF\;in\;the\;first\;60\;s}\times 100$$

When *MEF* in the first 60 s of dental work was compared with *MEF* in the next 60 s, a negative value was obtained when the value of *MEF* in the second 60 s was low, indicating the increase in muscle fatigue [23,24].

### 2.6. Statistical Analysis

IBM SPSS statistical Statistics for Windows, version 25 (IBM Corp., Armonk, NY, USA) was used to analyze all data (α = 0.05). First, the distribution of the data was investigated using the Shapiro–Wilk test; the data were not normally distributed. Therefore, Mann–Whitney U test was performed to compare the two types of dental UCS in EMG and muscle fatigue and to compare dental tasks (intraoral scanning and tooth preparation simulation). A Kruskal–Wallis H test was performed to compare the differences in EMG and muscle fatigue according to the muscles. The Bonferroni adjustment was performed for multiple comparisons.

## 3. Results

The mean working time was 444.7 ± 195.2 s for the tooth preparation task and 509.6 ± 142.6 s for the intraoral scanning task (1st: 571.5 ± 169.0 s, second: 496.3 ± 145.2 s, third: 461.0 ± 113.6 s). The time for the intraoral scanning task showed a significant decrease during the three repetitions (*p* < 0.001).

In both types of dental UCS, the *RMS EMG* of the tooth preparation task was higher than that of intraoral scanning, but there was no statistically significant difference (*p* = 0.147; Table 1). In addition, there was no significant difference between the muscle fatigue for the two types of simulations measured in the two types of dental UCS (*p* = 0.435; Table 2).

The intraoral scanning task and tooth preparation task showed a low risk level only in the SCM and a moderate risk level in other muscles (Table 1). During the intraoral scanning task, the digital integrated dental UCS showed significantly higher *RMS EMG* in the EDC and T (*p* < 0.001), while the conventional dental UCS showed significantly higher *RMS EMG* in the right T (*p* < 0.001; Table 1). During the tooth preparation task, both types of dental UCS showed significantly higher *RMS EMG* in the EDC, FDS, left SC, and T (*p* < 0.05; Table 1). There was also no significant difference between the *RMS EMG* with the two dental UCS (intraoral scanning task, *p* = 0.237; tooth preparation task, *p* = 0.543; Table 1).

In digital integrated dental UCS, there was no significant difference in muscle fatigue according to muscle in the intraoral scanning task (*p* = 0.138) and tooth preparation task (*p* = 0.219; Table 2). Similarly, in conventional dental UCS, there was no significant difference in muscle fatigue according to the muscle in the intraoral scanning task (*p* = 0.417) and tooth preparation task (*p* = 0.141; Table 2).

**Figure 3:**
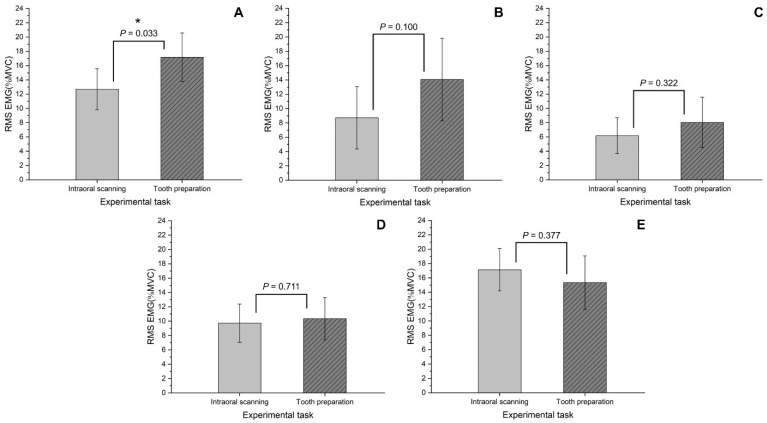
Comparison of *RMS EMG* (%*MVC*) according to the experimental task. (**A**) extensor digitorum communis; (**B**) flexor digitorum superficialis; (**C**) sternocleidomastoid muscle; (**D**) splenius capitis; (**E**) trapezius descendens.

**Figure 4:**
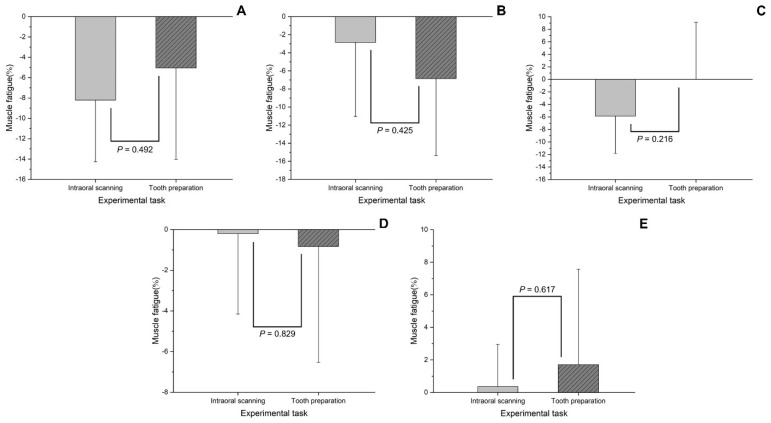
Comparison of muscle fatigue (%) according to the experimental task. (**A**) extensor digitorum communis; (**B**) flexor digitorum superficialis; (**C**) sternocleidomastoid muscle; (**D**) splenius capitis; (**E**) trapezius descendens.

When comparing the two tasks (intraoral scanning and tooth preparation), there was a significant difference in the *RMS EMG* of EDC (*p* = 0.033), and there was no significant difference in the *RMS EMG* and muscle fatigue between the two tasks in other muscles (*p* > 0.05). Both tasks showed moderate risk levels of *RMS EMG* in the T and EDC (Figure 3), and high muscle fatigue in the EDC and FDS (Figure 4).

Repeated use of the intraoral scanner three times did not show a significant change in *RMS EMG* (*p* = 0.639; Table 3) but showed a significant difference in muscle fatigue (*p* < 0.001; Table 4). In the FDS and SCM, using the intraoral scanner three times increased the muscle fatigue significantly (FDS, *p* = 0.043; SCM, *p* = 0.027; Table 4).

## 4. Discussion

The purpose of the present preliminary in vivo study was to evaluate muscle activation and fatigue in dentists during tooth preparation and intraoral scanning by performing simulations of the same with two types of dental UCS. The null hypothesis of our study was partially rejected (*p* > 0.05). There was no significant difference between muscle activity and fatigue with the two types of dental UCS (*RMS EMG*: *p* = 0.237 and *p* = 0.543; muscle fatigue: *p* = 0.228 and *p* = 0.287; Table 1 and Table 2), and there was no significant difference between muscle activity and fatigue with the two types of simulations (*RMS EMG*: *p* = 0.147; muscle fatigue: *p* = 0.435; Table 1 and Table 2). Repetitive learning of the intraoral scanner had no effect on muscle activity (*p* = 0.639; Table 3) but had a significant effect on muscle fatigue (*p* < 0.001; Table 4).

The learning effect (reduction in working time) according to repeated learning with the intraoral scanner has been confirmed in previous studies [26,27,28]. Similarly, in the present study, a significant decrease in the working time was observed with repetition of the intraoral scanning task (*p* < 0.001). In the previous study, the mean time of full-arch scanning using the intraoral scanner was reported to be 1255 s [29], but in the present study, the mean time was 509.6 s. This difference in scan time is due to rapid advances in intraoral scanners and the shift toward digital workflows. Although the task time was shortened, muscle activation was confirmed to be the same during the three repetitions due to the quantitative amount of the same task (*p* = 0.639; Table 3). However, contrary to the results of muscle activation, muscle fatigue showed significant accumulation after three repetitions (*p* < 0.001; Table 4); in particular, significant accumulation of muscle fatigue was confirmed in the arm (FDS: *p* = 0.043) and neck muscles (SCM: *p* = 0.027) after three repetitions (Table 4).

The weight of the intraoral scanner has been found to range from 113 g to 585 g [17]. In addition, because the manufacturing process of dental prostheses is being digitalized, the use of intraoral scanners is increasing. Therefore, considering the weight and increasing use of the intraoral scanner, it becomes necessary to evaluate muscle activation and fatigue. To the best of our knowledge, the present study is the first to evaluate this. The weight of an intraoral scanner used in the present study was 280 g. Our results suggest that continuous and repetitive intraoral scanning tasks should be avoided, and sufficient rest is important after an intraoral scanning task. In a previous study, a difference in muscle activation was observed with the type of muscle involved in performing the task [8,9,10]. Contrary to these results, a previous study reported that there were no significant differences in elbow or shoulder pain in 110 participants using either a light wide-handle curette or a narrow-handled heavy curette for scaling in 16 weeks [30].

In the present study, the intraoral scanning task and the tooth preparation task both showed a low risk level only in the SCM and a moderate risk level in the other muscles (Table 1). In the present study, high muscle activation was observed in the shoulder muscle (T) during the intraoral scanning task and in the two arm muscles (EDC and FDS) and in the shoulder muscle (T) in the tooth preparation task (Table 1). A previous study reported that a force of 0.9 N or more is applied to the teeth during tooth preparation for a desired shape [28]. Therefore, it can be inferred that the high activation of the arm muscles (EDC and FDS) during the tooth preparation task in the present study was because of gripping the dental ultra-fast handpiece and pressing it against the teeth (Figure 3). In addition, because the intraoral scanner is heavier than the high-speed dental handpiece [17], it can be inferred that the shoulder muscle (T) showed relatively high muscle activation during the intraoral scanning task compared to that during the tooth preparation task (Figure 3).

A previous study reported a difference in the neck muscle activation depending on the posture of the dentist when observing the oral cavity [8]. The posture for observing the oral cavity was corrected through the use of magnification lenses, and this lowered the activation of the neck muscles [8]. A previous study reported that the use of an ergonomic saddle and a dental magnifying glass improved working posture [31]. In a previous study, it was reported that the vision of an operator may accompany changes in the head and neck posture, which may affect the EMG [32]. In the present study, it was observed that activation of the neck muscle (SCM) increased during the intraoral scanning task compared with that during the tooth preparation task (Figure 3). This is because the intraoral scanning task is performed while observing a separate monitor while the scan is in progress, and the tooth preparation task is performed by bending the neck to observe the oral cavity (Figure 2). Muscle fatigue occurred regardless of the muscle type in both the intraoral scanning and tooth preparation tasks (Table 2). Therefore, it is important to note that activation of the neck muscles can be increased during the tooth preparation task [8], and sufficient rest is required after the task.

According to previous studies, various designs for dental UCS have been considered to help dentists provide treatment in the dental clinical environment [1,2,3]. In the present study, the design of the dental UCS had no effect on muscle activation and fatigue (*p* > 0.05; Table 1 and Table 2). Therefore, before performing each task, the participants adjusted the dentist’s chair and the patient’s chair according to their needs. Since both types of dental UCS used in the present study were adjusted for body type and convenience, it is presumed that the difference in dental UCS did not affect muscle activation and fatigue.

The present preliminary in vivo study has several limitations. First, the following variables were not considered during the simulation: postures, other than sitting, for treatment; various types of teeth involved in tooth preparation tasks; and types of high-speed dental handpieces and intraoral scanners. The mannequin used in the present study was difficult to reflect the patient’s oral environment. In actual clinical practice, the oral cavity does not remain fixed even if the patient cooperates. Moreover, the muscle tone associated with the presence of temporomandibular joint disorder can affect the degree of opening of the mouth, which can affect the dentist’s posture. This is a preliminary in vivo study, which has limitations in experimental configuration, and the findings should be further verified through additional studies. Second, although the sample size was determined by referring to a previous study [10], the present study included a small number of participants (six participants). In the present study, various factors were controlled for, and only participants who had a high willingness to participate, were very cooperative, and had a high understanding of its purpose were included. In addition, it is difficult for participants recruited in the present study to represent the results of various age and sex groups [33]. With increasing age, musculoskeletal disorders may increase, which may affect muscle fatigue and activation during certain activities. Finally, factors that may affect fatigue and muscle activation during work activities were not considered: subjective working positions, vision, practitioner parafunctions and bad habits, type of services performed, daily working hours, individual physical activity, degree of experience in the use of specific dental equipment. Conversely, a long-term clinical trial should be conducted by increasing the number of participants.

## 5. Conclusions

The difference between the two types of dental UCS did not affect muscle activation or fatigue. In addition, similar muscle activation and fatigue were observed during intraoral scanning and tooth preparation. However, in the present in vivo study, a moderate risk level of muscle activation was confirmed in the arm muscle (EDC) and shoulder muscle (T), and successive and repeated use of the intraoral scanner may have caused an increase in the muscle fatigue. Therefore, to reduce the occurrence of MSDs in dentists, it is recommended to take appropriate rest after performing continuous intraoral scanning and tooth preparation tasks. In addition, further studies are needed considering the number of participants and factors affecting fatigue and muscle activation during work activities.

## Figures and Tables

**Table 1:** Comparison of mean *RMS EMG* (%*MVC*) according to muscle type and dental unit chair system.

Muscle Type	Intraoral Scanning Task			Tooth Preparation Task		
	Dental Unit Chair System		*p* *	Dental Unit Chair System		*p* *
	Integrated	Conventional		Integrated	Conventional	
Extensor digitorum communis	13.6 ± 4.0 ^ac^	11.7 ± 2.5 ^a^	0.368	16.4 ± 4.8 ^ab^	17.9 ± 6.9 ^ab^	0.668
Flexor digitorum superficialis	10.5 ± 5.5 ^ab^	5.5 ± 3.2 ^a^	0.209	15.4 ± 8.0 ^ab^	12.7 ± 4.3 ^ab^	0.487
Left sternocleidomastoid muscle	8.5 ± 6.2 ^ab^	6.0 ± 3.2 ^a^	0.409	9.4 ± 5.2 ^a^	9.3 ± 6.9 ^a^	0.967
Right sternocleidomastoid muscle	4.6 ± 2.5 ^b^	5.3 ± 1.3 ^a^	0.551	5.5 ± 3.9 ^a^	7.7 ± 6.2 ^a^	0.490
Left splenius capitis	9.4 ± 4.8 ^ab^	12.0 ± 4.9 ^a^	0.397	11.8 ± 4.6 ^ab^	12.8 ± 4.5 ^ab^	0.712
Right splenius capitis	10.2 ± 4.0 ^ab^	7.1 ± 3.8 ^a^	0.207	8.8 ± 4.1 ^a^	7.7 ± 4.5 ^a^	0.675
Left trapezius descendens	17.0 ± 4.4 ^ac^	11.1 ± 5.6 ^a^	0.077	14.2 ± 6.5 ^ab^	10.6 ± 4.3 ^ab^	0.298
Right trapezius descendens	19.5 ± 5.7 ^c^	20.7 ± 7.7 ^b^	0.755	20.3 ± 8.8 ^b^	16.1 ± 9.4 ^ab^	0.451
Mean	11.7 ± 6.3	10.1 ± 6.3		12.7 ± 7.1	11.8 ± 6.7	
*p* **	<0.001	<0.001		0.003	0.042	
*p* ***	0.237			0.543		
*p* ****	0.147					

Significance was determined by the Mann–Whitney U test (*, comparison according to unit chair system in each muscle; ***, comparison of unit chair systems in overall mean; and ****, comparison of two simulations), *p* < 0.05. **, Significance determined by Kruskal–Wallis H test (comparison of each muscle), *p* < 0.05. RMS, root mean square; EMG, Electromyography.

**Table 2:** Comparison of mean muscle fatigue (%) according to muscle type and dental unit chair system.

Muscle Type	Intraoral Scanning Task			Tooth Preparation Task		
	Dental Unit Chair System		*p* *	Dental Unit Chair System		*p* *
	Integrated	Conventional		Integrated	Conventional	
Extensor digitorum communis	−6.7 ± 3.4	−9.6 ± 9.0	0.488	−2.8 ± 5.1	−7.2 ± 14.8	0.513
Flexor digitorum superficialis	−4.4 ±6.6	−1.2 ± 10.7	0.554	−4.2 ± 9.4	−9.4 ± 13.3	0.455
Left sternocleidomastoid muscle	−17.8 ± 7.2	−0.9 ± 18.0	0.058	−17.0 ± 19.0	−3.9 ± 6.2	0.142
Right sternocleidomastoid muscle	−8.0 ± 11.9	3.4 ± 12.1	0.126	15.3 ± 42.4	5.7 ± 16.3	0.623
Left splenius capitis	8.7 ± 9.9	−2.9 ± 4.2	0.033	2.9 ± 11.1	−11.2 ± 10.4	0.047
Right splenius capitis	−7.2 ± 9.7	0.6 ± 5.8	0.127	3.2 ± 8.4	3.5 ± 11.7	0.960
Left trapezius descendens	3.2 ± 11.1	−3.4 ± 3.4	0.190	3.2 ± 16.7	1.7 ± 5.7	0.847
Right trapezius descendens	−0.3 ± 5.0	1.9 ± 4.3	0.407	3.4 ± 7.8	−3.4 ± 10.8	0.240
Mean	−4.0 ± 11.0	−1.5 ± 9.7		0.5 ± 19.3	−3.0 ± 12.3	
*p* **	0.148	0.417		0.219	0.141	
*p* ***	0.228			0.287		
*p* ****	0.435					

Significance was determined by the Mann–Whitney U test (*, comparison according to unit chair system in each muscle; ***, comparison of unit chair systems in overall mean; and ****, comparison of two simulations), *p* < 0.05. **, Significance determined by Kruskal–Wallis H test (comparison of each muscle), *p* < 0.05.

**Table 3:** Comparison of mean *RMS EMG* (%*MVC*) in the first, second, and third repetitions of the intraoral scanning task.

Muscle Type	Trial No.			*p* **	*p* ***
	1	2	3		
Extensor digitorumcommunis	11.9 ± 2.4 ^a^	11.7 ± 3.1 ^ab^	13.2 ± 3.3 ^ab^	0.656	0.639
Flexor digitorumsuperficialis	5.6 ± 2.7 ^b^	6.5 ± 3.0 ^b^	6.9 ± 3.2 ^b^	0.765	
sternocleidomastoid muscle	5.9 ± 1.0 ^b^	5.7 ± 2.8 ^b^	7.7 ± 3.8 ^b^	0.434	
splenius capitis	10.8 ± 4.7 ^ab^	8.9 ± 3.2 ^b^	8.9 ± 4.0 ^b^	0.661	
trapezius descendens	15.5 ± 4.7 ^a^	15.9 ± 5.5 ^a^	18.1 ± 5.4 ^a^	0.653	
*p* *	<0.001	<0.001	<0.001		

The same superscript lowercase letters (column) are not significantly different according to the Mann–Whitney U-test and Bonferroni correction method. Significance was determined by the Kruskal–Wallis H test (*, comparison of each muscle; **, comparison of task repetitions in each muscle; and ***, comparison of task repetitions overall); *p* < 0.05. RMS, root mean square; EMG, Electromyography.

**Table 4:** Comparison of intraoral scanning task mean muscle fatigue (%) in the first, second, and third repetitions.

Muscle Type	Trial No.			*p* **	*p* ***
	1	2	3		
Extensor digitorumcommunis	−5.3 ± 8.8	−10.4 ± 10.7 ^a^	−11 ± 7.1	0.509	<0.001
Flexor digitorumsuperficialis	1.9 ± 17.2 ^A^	0.9 ± 8.1 ^abA^	−16.8 ± 12.2 ^B^	0.043	
sternocleidomastoid muscle	−2.3 ± 9.3 ^A^	2.1 ± 6.7 ^bA^	−10.5 ± 5.2 ^B^	0.027	
splenius capitis	−1.7 ± 6.9	0.1 ± 3.5 ^ab^	−3.8 ± 3.5	0.406	
trapezius descendens	−0.4 ± 6.5	−0.5 ± 4.6 ^ab^	−4.6 ± 5.4	0.351	
*p* *	0.814	0.041	0.066		

The same superscript lowercase letters (column) and same superscript uppercase letters (row) are not significantly different according to the Mann–Whitney U-test and Bonferroni correction method. Significance was determined by the Kruskal–Wallis H test (*, comparison of each muscle; **, comparison of task repetitions in each muscle; and ***, comparison of task repetitions overall); *p* < 0.05.

## Data Availability

The datasets used and/or analyzed during the current study are available from the corresponding author on reasonable request.
